# Risk factors for adverse perinatal outcomes in imprisoned pregnant women: a systematic review

**DOI:** 10.1186/1471-2458-5-111

**Published:** 2005-10-17

**Authors:** Marian Knight, Emma Plugge

**Affiliations:** 1 National Perinatal Epidemiology Unit, University of Oxford, Oxford, UK; 2 Department of Public Health, University of Oxford, Oxford, UK

## Abstract

**Background:**

Imprisoned pregnant women constitute an important obstetric group about whom relatively little is known. This systematic review was conducted to identify the risk factors associated with adverse pregnancy outcome present in this group of women.

**Methods:**

The review was conducted according to a prespecified protocol. Studies of any design were included if they described information on any of the pre-specified risk factors. We calculated the results as summary percentages or odds ratios where data was available on both cases and population controls.

**Results:**

The search strategy identified 27 relevant papers of which 13 met the inclusion criteria, involving 1504 imprisoned pregnant women and 4571 population control women. Imprisoned women are more likely to be single, from an ethnic minority, and not to have completed high school. They are more likely to have a medical problem which could affect the pregnancy outcome and yet less likely to receive adequate antenatal care. They are also more likely to smoke, drink alcohol to excess and take illegal drugs.

**Conclusion:**

Imprisoned women are clearly a high risk obstetric group. These findings have important implications for the provision of care to this important group of women.

## Background

Although women make up only a small proportion of the 9 million people imprisoned worldwide [1], their numbers are increasing rapidly and consistently across a number of countries [2,3]. For example, the number of women imprisoned in England and Wales has risen almost threefold over the past decade [3]. Most of these women will be of childbearing age and an estimated 6% of imprisoned women are pregnant [3,4]. This implies that in England and Wales alone there are about 240 pregnant women in prison at any one time, and in the United States of America over 6,000.

These women constitute an important obstetric group about whom relatively little is known. Available evidence suggests that they are more likely to come from socially deprived backgrounds and to smoke, drink alcohol to excess and abuse illegal drugs than the general population [5-10]. However, estimates of the prevalence of these risk behaviors in this population vary. These factors may affect both the health of the women themselves and also their offspring and are therefore of considerable public health significance. It is important to recognize these factors in order to allow appropriate planning of future services for this increasing number of women. The objective of this study therefore was to identify the risk factors associated with adverse pregnancy outcome present in imprisoned women through a systematic review of the literature.

## Methods

The review was conducted according to a pre-specified protocol. We identified possible risk factors for poor perinatal outcomes from guidelines produced by the American College of Obstetricians and Gynecologists (ACOG) [11] and the National Institute for Clinical Excellence (NICE) [12], and literature review. We used multiple strategies to identify relevant articles, searching for any studies published from 1980 up to the end of May 2004. We searched Medline, Embase, CINAHL, Psycinfo, and the Cochrane library database. We identified search terms from database thesauri (indicated by italics) and terms were also included as free text. We used a combination of terms relating to pregnancy (e.g. pregnan*, *pregnancy, pregnancy-outcome, pregnant-women*) and to imprisonment (e.g. prison*, gaol*, jail*, incarcerat*, *prisons, prisoners*) combining them using Boolean operators. We also carried out hand searches of the references of selected papers and relevant policy documents. We identified grey literature and unpublished research by searching the National Research Register, the NHS Centre for Reviews and Dissemination database and the internet (accessed May 2004 using Google search engine).

We included studies if they described any of the pre-specified characteristics of pregnant women (Table 1) who were imprisoned at any stage during their pregnancy in any category of prison. No restrictions were placed on the study design that could be included nor on the basis of the language of publication or the geographical location of the study. Non-English articles were translated. We excluded studies which did not include information on the pre-specified risk factors. An assessment of methodological quality was made according to the principles recommended for assessing non-experimental studies in the Cochrane Reviewers Handbook [13]. Potential for selection, performance, attrition and detection bias was assessed. Studies were graded for quality as A, B or C indicating a low, moderate or high risk of bias respectively.

**Table 1:** Risk factors for poor perinatal outcomes pre-specified in the study protocol

**Risk factors**	
Age over 40	
Age less than 18	
Primiparity	
Parity >4	
Previous preterm delivery or midtrimester loss	
Previous stillbirth or neonatal death	
Previous infant with a congenital anomaly	
Smoking	
Alcohol use in pregnancy	
Illicit drug use	
Maternal medical problem known to be associated with poor perinatal outcome:	epilepsy
	diabetes
	autoimmune disorder
	HIV
	hypertension
	cardiac disease
	renal disease
Previous low birth weight infant	
Non-white race	
Educational level (Not completed high school/ A levels)	
Inadequate antenatal care (first antenatal visit in second trimester or beyond, or fewer than six visits in total)	
Single marital status	
Body Mass Index (BMI) >35	
BMI <18	
Previous Cesarean section	

Two investigators independently extracted the data according to a fixed protocol. Differences were resolved by discussion. As well as the pre-specified risk factors, we also collected data on study design, case selection, control selection, nature of the control group and location of the study. We expressed the frequencies of risk factors for poor perinatal outcomes among imprisoned women as percentages. Where data was available on both cases and population controls who were not matched for a particular risk factor, odds ratios were calculated using a fixed effects model (Mantel-Haenszel). The X^2 ^test for heterogeneity was used to assess the extent to which the results of the studies were in agreement.

## Results

We identified 27 relevant papers. Of these, 13 met the inclusion criteria (Table 2), the majority of which were conducted in the USA. All were written in English with the exception of one German paper which was translated. The excluded papers were either discussion documents or did not contain any data on the pre-specified risk factors. The included studies comprise a total of 1504 imprisoned pregnant women and 4571 population control women.

**Table 2:** Included studies

**Reference**	**Study Design**	**Setting**	**Participants (imprisoned pregnant women and population control women)**	**Quality**
Stauber 1984 [14]	Cohort	Berlin, Germany	43 pregnant women imprisoned between 1973 and 1982. 172 women from the same hospital matched with cases by age, parity, marital status and year of birth.	B
Elton 1985 [15]	Cohort	Manchester, UK	298 pregnant women admitted to one prison 1975–1982. 298 non-imprisoned women selected from the same hospital antenatal clinic matched with cases by age, marital status, previous stillbirths and height.	A
Shelton 1989 [16]	Case series	Missouri, Maryland, USA	26 imprisoned women who delivered in 1982.	B
Cordero 1991 [9]	Case Series	Ohio, USA	53 pregnant women imprisoned for between 1 week and 90 days. 53 matched pregnant women imprisoned for greater than 120 days. 1986–1990.	A
Cordero 1992 [8]	Case Series	Ohio, USA	233 pregnant women imprisoned in the state medium-security prison 1986–1990.	B
Egley 1992 [7]	Cohort	North Carolina, USA	69 imprisoned pregnant women cared for at one hospital during 1988. 69 non-imprisoned pregnant women from the same hospital matched with cases by age, race, parity and date of entry into prenatal care.	A
Fogel 1993 [6]	Case Series	North Carolina, USA	89 pregnant women imprisoned between 1986 and 1989.	A
Terk 1993 [10]	Cohort	Texas, USA	76 imprisoned pregnant women 1987–1990. 117 unmatched randomly-chosen non-imprisoned pregnant women from the same hospital during the same time period.	A
Martin 1997 [17]	Cohort	North Carolina, USA	168 imprisoned pregnant women who gave birth to one infant between 1988 and 1991 identified from state records. 3910 unmatched randomly selected women resident in and delivering in North Carolina over the same time period.	B
Kyei-Aboagye 2000 [5]	Cohort	Massachusetts, USA	31 imprisoned pregnant women delivering at one hospital between 1993 and 1996. 71 unmatched randomly chosen non-imprisoned women delivering at the same hospital.	B
Mertens 2001 [18]	Cohort	Illinois, USA	71 pregnant women imprisoned in a county jail in one calendar year. 51 pregnant women identified from state records and matched with cases by age, race, gravidity and zip code of residence.	C
Siefert 2001 [19]	Case Series	Michigan, USA	120 pregnant women imprisoned before commencement of a residential program (1987–1991), 44 unmatched pregnant women imprisoned after the residential program (1991–1995).	A
Barkauskas 2002 [20]	Case Series	Michigan, USA	90 imprisoned pregnant women in a residential care program and 40 unmatched imprisoned pregnant women not in the residential programme.1996–1998.	C

The risk factor profile of imprisoned pregnant women is summarized in Table 3. We did not identify any studies which described the proportion of imprisoned pregnant women who were aged less than 18, who had a parity greater than four, who had had a previous infant with a congenital anomaly, who had a body mass index (BMI) greater than 35 or less than 18, or who had had a previous caesarean section. Five of the risk factors were identified to be present in more than 50% of imprisoned pregnant women: single marital status (83.7%), non-white race (66.7%), smoking (66%), low educational attainment (54.4%) and illicit drug use (53.7%).

**Table 3:** Risk factor profile of imprisoned pregnant women

**Risk factor**	**Number of women studied**	**Number of studies**	**Percentage of imprisoned pregnant women with identified risk factor**
Single marital status	997	7	**83.7**
Non-white race	1042	10	**66.7**
Smoking	838	8	**66.0**
Educational level (not completed high school/ A levels)	529	6	**54.4**
Drug use	646	7	**53.7**
Inadequate prenatal care	704	6	**30.5**
Primiparity	944	8	**26.7**
Previous low birth weight infant	88	1	**25.0**
Alcohol use	363	4	**19.8**
Previous preterm delivery or mid-trimester loss	428	3	**15.9**
Maternal medical problem	100	2	**11.0**
Age over 40	233	1	**2.6**
Previous stillbirth or neonatal death	298	1	**1.3**

**Figure 1:**
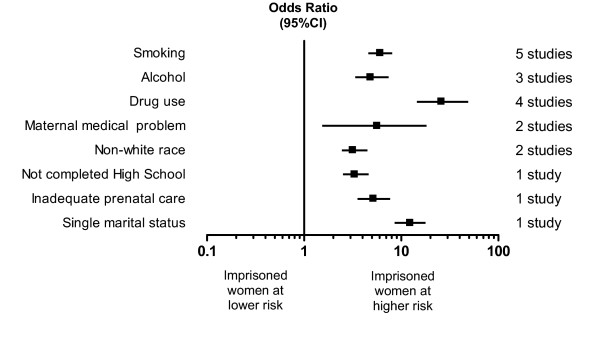
Summary odds ratios for the predefined risk factors for poor perinatal outcomes of imprisoned women compared to controls. Figures greater than 1 indicate risk factors occurring more frequently in imprisoned pregnant women than population control pregnant women.

Eight outcomes were reported in studies which included an appropriate population control group of non-imprisoned pregnant women, enabling summary odds ratios to be calculated. These are shown in Figure 1.

Five studies reported smoking status in imprisoned women and controls [5,7,10,14,17] (386 imprisoned, 4339 control women). Imprisoned women were significantly more likely to smoke during pregnancy than control women (OR 6.05 (95% CI 4.74–7.73). Imprisoned women were also significantly more likely to use alcohol during pregnancy (3 studies [5,10,17], 274 imprisoned, 4098 control women, OR 4.82 (3.23–7.19)) and to use illicit drugs (4 studies [5,7,10,14], 218 imprisoned, 429 control women, OR 25.86 (14.06–47.57)). It should be noted, however, that there was significant heterogeneity between the studies reporting alcohol use (X^2 ^= 11.56, p = 0.003). This heterogeneity was entirely due to one small study [5] (weight in meta-analysis 2.78%, OR 54.63 (11.24–265.4)). It was not clear from this paper how alcohol use in control women was assessed, which may have resulted in performance bias in this study. The remaining included studies reported smaller, but still significant, increased use of alcohol in imprisoned pregnant women compared with the non-imprisoned group.

Only two studies reported maternal medical problems in imprisoned women and controls [5,7] (100 imprisoned, 140 control women). Imprisoned pregnant women were significantly more likely to have medical problems likely to impact on pregnancy outcome than control women (OR 5.64 (1.66–19.11). We identified only two studies which reported race in unmatched imprisoned women and controls [5,10] (244 imprisoned, 4027 control women); these studies showed that imprisoned women were significantly more likely to be of non-white race (OR 3.17 (2.39–4.19) than control women.

The remaining three outcomes were reported in imprisoned and control women in only one study [17] (168 imprisoned, 3910 control women). This study showed that imprisoned pregnant women were more likely not to have completed high school than control women (OR 3.30 (2.42–4.51)), were more likely to be of single marital status (OR 12.32 (8.21–18.50)), and were more likely to have received inadequate prenatal care (OR 5.15 (3.60–7.38).

## Discussion

This is the first review to describe in detail the risk factor profile of imprisoned pregnant women. The results show that these women are clearly a group at high risk of poor perinatal outcomes. They are more likely to be single, from an ethnic minority (predominantly African-American and Hispanic), and not to have completed high school. They are more likely to have a medical problem which could affect the pregnancy outcome and yet less likely to receive adequate antenatal care. They are also more likely to smoke, drink alcohol to excess and take illegal drugs. These findings have implications for the provision of care to this important group of women.

Overall, more than 30% of imprisoned pregnant women received inadequate prenatal care, classified by the date of their first prenatal visit or by the total number of visits. Clearly, this inadequacy of care may relate to the period of pregnancy before these women were imprisoned, and it is therefore important to undertake work to ensure that prenatal care is available and accessible to the socially disadvantaged populations from which these women come. However, it also remains a priority to ensure that provision for prenatal care is adequate in all prison settings so that these women and their unborn infants are not further compromised by poor care during imprisonment.

The results of this review also indicate areas of prenatal care for imprisoned pregnant women on which there needs to be a specific focus. 66% of imprisoned pregnant women smoke, nearly 20% use alcohol to excess and over 50% use illegal drugs. This shows a clear need to provide programs particularly for pregnant drug users to assist not only with cessation of use during pregnancy as appropriate, but also with long-term drug-free maintenance once women have completed their pregnancy and period of imprisonment.

This review also provided some limited evidence that imprisoned pregnant women are more likely to suffer from medical problems known to be associated with poor perinatal outcomes, such as diabetes, epilepsy, hypertension, cardiac or renal disease. Prison prenatal services need therefore to be sufficiently flexible to allow for more specialist care for women with particular problems.

We were not able to extract information on a number of factors known to increase the risk of poor perinatal outcomes, for example over- and underweight, grand-multiparity and previous caesarean section. Although several studies included some information on weight, there were no consistent definitions used and no studies reported body mass index (BMI). Obesity is known to be associated with a range of adverse perinatal outcomes [21], and population prevalence is known to be increasing [22,23]. This is therefore an important risk factor to identify in imprisoned pregnant women. Previous cesarean section is also known to be associated with adverse perinatal outcomes [24], and identification of this factor in imprisoned pregnant women would give a more comprehensive picture of the pattern of risk in this group.

The results highlight the importance of pregnant prisoners as a high risk population. This has important policy implications. Clearly the level of provision of services for pregnant women in prison should not be based on that provided for the general population of pregnant women – need is much greater in the prisoners. Furthermore, there is some evidence to suggest that imprisonment might actually have a beneficial effect on particular pregnancy outcomes; limited research on the pregnancy outcomes of imprisoned women has shown that prison actually improves fetal outcomes and the longer spent in prison, the better the outcome [15,17]. The authors give a number of possible reasons for this; prison provides food, shelter and protection from abusive partners; it ensures access to antenatal care and moderates the use of alcohol and drugs. This suggests that appropriate health service provision for pregnant women in prison can be effective and should be prioritised in the health planning process. Clearly more research is needed in this area.

Undoubtedly there are problems with the generalisability of these findings. Most of the studies come from the USA although a few come from the UK and one from Germany. The demographics of female prison populations vary considerably across the world but tend to reflect the composition of the poorest, most disadvantaged sections of society from which they come. Thus whilst the prevalence of risk factors for adverse perinatal outcomes may show some variation from country to country, it is unlikely that in any country the prevalence is lower in the prison population. It is likely that pregnant prisoners have great health needs wherever they are from. Health planners and providers in all countries should not ignore this important population. Furthermore although some studies identified were conducted more than twenty years ago, we would argue that rates of smoking etc. have declined more slowly in disadvantaged groups and therefore are unlikely to have changed substantially from the figures reported.

Although this review encompasses information on more than 1500 pregnant women and 4500 ontrols, the number of studies identified were relatively few and we did not exclude any on the basis of quality. Of the thirteen studies identified, only 6 were assessed to be of high quality (low risk of bias), 5 were of moderate quality (moderate risk of bias), and 2 of low quality (high risk of bias). Because of the small number of studies reporting similar risk factors, we were unable to perform a sensitivity analysis to investigate the effect of study quality on the reported results. Thus the potential for bias needs to be taken into account when considering the overall results of the review.

The small study numbers have also impacted on the precision of some of the results. The confidence intervals for the odds ratios associated with drug use, a maternal medical problem and single marital status are wide. However, all are significantly greater than 1 indicating that these risk factors are present to a greater extent in imprisoned pregnant women and must be taken into account when planning services.

## Conclusion

More research could be undertaken to describe more comprehensively the risk factors for poor perinatal outcomes in this group of women. However, it is clear from this review that the factors already identified are likely to impact significantly on both the health of imprisoned pregnant women and their infants. Imprisoned pregnant women are a socially disadvantaged group and prison presents an opportunity to engage with them effectively and meet their substantial needs. It is necessary to ensure the provision of adequate, tailored prenatal services for these women in order to prevent future maternal and perinatal morbidity and mortality.

## List of abbreviations

ACOG American College of Obstetricians and Gynecologists

BMI Body Mass Index

NICE National Institute for Clinical Excellence

OR Odds Ratio

## Competing interests

The author(s) declare that they have no competing interests.

## Authors' contributions

MK and EP drafted the report and were involved in study design, data extraction, statistical analysis and interpretation of the results. Both authors read and approved the final manuscript.

## Pre-publication history

The pre-publication history for this paper can be accessed here:


